# Nimotuzumab Enhances the Radiosensitivity of Cancer Cells In Vitro by Inhibiting Radiation-Induced DNA Damage Repair

**DOI:** 10.1371/journal.pone.0070727

**Published:** 2013-08-16

**Authors:** Yuan-yuan Qu, Song-liu Hu, Xiang-ying Xu, Rui-zhi Wang, Hong-yang Yu, Jian-yu Xu, Lin Chen, Guang-lu Dong

**Affiliations:** 1 Department of Radiation Oncology, the Third Affiliated Hospital of Harbin Medical University, Harbin, Heilongjiang, China; 2 Heilongjiang Province Institute of Cancer Research, Harbin, Heilongjiang, China; 3 Department of Radiation Oncology, the Second Affiliated Hospital of Harbin Medical University, Harbin, Heilongjiang, China; University of Kentucky, United States of America

## Abstract

**Background:**

Nimotuzumab is a humanized IgG1 monoclonal antibody specifically targeting EGFR. In this study, we aimed to investigate the molecular mechanisms of nimotuzumab in its effects of enhancing cancer cell radiosensitivity.

**Principal Finding:**

Lung cancer A549 cells and breast cancer MCF-7 cells were pretreated with or without nimotuzumab for 24 h before radiation to perform the clonogenic survival assay and to analyze the cell apoptosis by flow ctyometry. γ-H2AX foci were detected by confocal microscopy to assess the effect of nimotuzumab on radiation induced DNA repair. EGFR activation was examined and the levels of DNA damage repair related proteins in A549 cells at different time point and at varying doses exposure after nimotuzumab and radiation treatment were examined by Western blot. Pretreatment with nimotuzumab reduced clonogenic survival after radiation, inhibited radiation-induced EGFR activation and increased the radiation-induced apoptosis in both A549 cells and MCF-7 cells. The foci of γ-H2AX 24 h after radiation significantly increased in nimotuzumab pretreated cells with different doses. The phosphorylation of AKT and DNA-PKcs were remarkably inhibited in the combination group at each dose point as well as time point.

**Conclusions:**

Our results revealed that the possible mechanism of nimotuzumab enhancing the cancer radiosensitivity is that nimotuzumab inhibited the radiation-induced activation of DNA-PKcs through blocking the PI3K/AKT pathway, which ultimately affected the DNA DSBs repair.

## Introduction

Radiotherapy plays a major role in treating multiple cancers with curative or palliative intention. Approximately 50% of patients suffering with cancers need radiotherapy throughout their treatment process. However, the disease control and survival rate of patients who receive radiotherapy alone or in combination with chemotherapy remain dismally low. Traditional cytotoxic agents with radiosensitizing function often simultaneously increase normal tissue toxicity, which limits their clinical application when combined with radiotherapy. Recently, therapies targeting epidermal growth factor receptor (EGFR) have exhibited excellent anticancer effects with mildly adverse effects and significantly enhanced cancer radiosensitivity in preclinical and clinical studies [1], [2]. EGFR targeted therapies combined with radiotherapy has been regarded as a very potential strategy for treatment of some cancers of epithelial origin.

EGFR targeted therapies consist mainly of two approaches: 1) monoclonal antibodies (mAb) that target the extracellular domain of the receptor in the ligand-binding region, namely cetuximab, nimotuzumab and panituzumab; or 2) small molecules that inhibit EGFR's intracellular tyrosine kinase activity, such as gefitinib and erlotinib [3]. Most of these agents have been extensively studied *in vitro* and *in vivo* in their capacity of enhancing tumor radiosensitivity. By blocking EGFR activation and its downstream signaling, such as the PI3K-AKT and RAS-MAPK pathways, these anti-EGFR agents enhance the cytotoxic effect of ionizing radiation by inducing cell cycle arrest and apoptosis and inhibiting cell proliferation, metastasis and tumor angiogenesis [4], [5].

Nimotuzumab is a humanized IgG1 monoclonal antibody that blocks EGF, TGF-α and other ligands from binding to EGFR, as well as hindering the receptor from exposing its dimerization motif [6]. Nimotuzumab attaches to EGFR with moderate binding affinity (Kd: 4.5×10^−8^ m) compared with cetuximab, which has a binding affinity of more than 10 fold higher [6]. Studies *in vitro* have shown that nimotuzumab binds bivalently (i.e., with both antibody arms to two targets simultaneously) to EGFR with moderate or high density, which is the stable pattern of attachment [7], [8]. In normal tissues with low EGFR density, nimotuzumab has less affinity and binds EGFR with less avidity, which spares the normal tissues, including skin and mucosa, from severe cytotoxicity. This explains why nimotuzumab is characterized by slight treatment-related toxicities in clinical application while displaying similar or superior anticancer effects as compared to other anti-EGFR monoclonal antibodies. As a promising therapeutic monoclonal antibody, nimotuzumab combined with radiation is being studied extensively in its efficacy of treating cancers of epithelial origin.

Nimotuzumab has been proven to selectively enhance antitumor effects of ionizing radiation of NSCLC cell lines with high EGFR expression [9]. In addition, an in vivo study in mice xenografts transplanted with a glioma cell line showed that both nimotuzumab and cetuximab increased radiosensitivity of the transplanted subcutaneous tumors [10]. In phase II/III clinical trials, nimotuzumab combined with radiotherapy has achieved excellent outcome in treating locally advanced head and neck cancers [11], [12]. It is reported that cetuximab inhibits radiation-induced EGFR nuclear translocation, and this process is associated with the suppression of DNA-PKcs activity [13], [14]. Other studies have shown that tyrosine kinase inhibitors enhance radiosensitivity by suppressing cellular capacity of radiation-induced DNA-damage repair [15], [16]. These results indicate that therapeutic monoclonal antibody treatment combined with radiation therapy may impact the radiation-induced DNA damage response. However, the underlying mechanisms by which nimotuzumab functions in radiosensitization still remain elusive. In this study, using two cultured cancer cell lines, we aimed to investigate potential molecular mechanism of nimotuzumab in enhancing cellular radiosensitivity of cancers.

## Materials and Methods

### Cells, cell culture and reagents

The human NSCLC cell line A549 and breast cancer cell line MCF-7 (provided by Heilongjiang Province institute of cancer research, Harbin, China) were maintained in RPMI 1640 (GIBCO) medium supplemented with 10% fetal bovine serum (FBS) (NQBB, Australia) under a humidified atmosphere of 5% CO2 at 37°C. Nimotuzumab was provided by Biotech Pharmaceutical Co. Ltd (Beijing, China).

### Clonogenic survival assay

Exponentially growing cells in 25 cm^2^ flasks were trypsinized, harvested, and counted. They were diluted serially to appropriate densities, and plated in triplicate with certain numbers (different cell numbers according to the dose irradiated. For example, 200 cells per flask for 2Gy, 500 cells for 4Gy, 1000 cells for 6Gy, 4000 cells for 8Gy, 10000 cells for 10Gy and 100 cells for control.) in 25 cm^2^ flasks containing 5 ml of culture medium in the absence or presence of 700 nM nimotuzumab. Twenty-four hours after incubation, the cells were exposed to 2, 4, 6, 8 and 10 Gy of 4MV X-ray generated by a high energy linear accelerator (Elekta synergy, Stockholm, Sweden) at a dose rate of 3 Gy min^−1^. The cells were then washed with phosphate-buffered saline (PBS) and cultured in drug-free medium for 10–14 days to form colonies. After that, the colonies were fixed with methanol∶acetic acid (10∶1, v/v), and then stained with crystal violet. Colonies containing ≥50 cells were counted. The surviving fraction was calculated as: (mean number of colonies)/(number of inoculated cells×plating efficiency). Plating efficiency was defined as: (mean number of colonies)/(the number of inoculated cells for control, which were not exposed to radiation with or without nimotuzumab). The data were fitted into the classic single-hit multi-target model: SF = 1−(1−e^−D/D0^)^ N^ to draw the dose- survival curve, from which the parameters (D0, Dq, N and SF2) representing the intrinsic cellular radiosensitivity were derived. The sensitivity enhancement ratio (SER) was calculated as: (D_0_ of radiation treated cells/D_0_ of nimotuzumab combined with radiation treated cells).

### Cell apoptosis analysis

Cells treated with or without 700 nM nimotuzumab for 24 h were exposed to different doses of radiation (0, 2, or 8 Gy), and were harvested at 48 h after radiation for cell apoptosis analysis. Approximately 1×10^6^ cells in each group were stained with annexin V-FITC and propidium iodide (PI) for apoptosis analysis according to manufacturer's instruction (Beyotime, China). Cells were then analyzed by fluorescence-activated cell sorting (FACS, calibur, BD, US).

### γ-H2AX foci detection by confocal microscopy

Cells grown on coverslips in six-well plates were treated with or without 700 nM nimotuzumab for 24 h. Subsequently, cells were radiated at varying doses (1, 2, 4 or 8 Gy) followed by incubation for 24 h. Afterwards, cells were fixed with ice cold 70% ethanol for 30 min, washed 3 times with PBS and blocked with 3% BSA and 0.1% Triton-X100 in PBS for 30 min. After removing the blocking buffer, cells were incubated with 2–4 ug/ml γ-H2AX antibody conjugated with FITC (Millipore, Billerica, MA) in blocking buffer for 2 h in the dark. The excessive antibodies were washed away by PBS. Finally, the 24 h residual γ-H2AX was examined by confocal microscopy (ZEISS, LSM700, Germany). For each data point at least 300 nuclei were evaluated.

### Detection of EGFR phosphorylation and DNA damage repair related proteins by Western-blot

A549 cells and MCF-7 cells were treated with or without 700 nM nimotuzumab for 24 h and were radiated with 4 Gy X-ray to detect the EGFR phosphorylation. Treated cells were harvested at 2 h after radiation. A549 cells treated as described above were harvested at the 0.5, 1, 2, or 6 h after radiation for dynamic analysis of DNA damage repair related proteins. Same amounts of A549 cells with the same treatment schedule were irradiated with different dose (0, 1, 2, 4, 6Gy), incubated for 2 hours, then were harvested. Subsequently, cell pellets were treated with lysis buffer (Beyotime, China), 1 mM PMSF (Beyotime, China) and 1 protease inhibitor cocktail tablet (Roche applied science, Mannheim, Germany) per 10 ml solution was added, and total proteins were isolated. Equal amounts of proteins (30 µg) were separated on 8% SDS-PAGE gels, and then transferred to PVDF membranes. The membranes were blocked with 5% non-fat dry milk in TBS-T20 for 1 h at room temperature and subsequently incubated with primary antibodies overnight at 4°C. After washing three times with TBST, the membranes were incubated with horseradish peroxidase–conjugated secondary antibodies (Zhongshan, China) for 1 h. The blots were developed by chemiluminescence detection kit for HRP (BI, Isreal) and visualized with a chemiluminescence imaging system (FLuorchemFC2, Alpha Innotech, US).

The primary antibodies were diluted as follow: anti-EGFR and anti-pEGFR (Cell Signaling Technology, Denvers, MA), 1∶1000; anti-DNA-PK (Cell Signaling Technology, Denvers, MA), 1∶1000; anti-pDNA-PK (Abcam, Cambridge, UK), 1∶500; anti-Ku70 (Epitomic, Burlingame, Calif), 1∶4000; anti-ATM (Cell Signaling Technology, Denvers, MA), 1∶1000; anti-pATM (Cell Signaling Technology, Denvers, MA), 1∶1000; anti-AKT (Cell Signaling Technology, Denvers, MA), 1∶1000; anti-pAKT(Cell Signaling Technology, Denvers, MA), 1∶1000; anti-γ-H2AX (Cell Signaling Technology, Denvers, MA), 1∶1000; and anti-β-actin (Zhongshan, Beijing, China), 1∶2000.

### Statistical analysis

Data were analyzed using paired t-test's and described as mean ±SD. A difference was regarded as significant if P<0.05.

## Results

### Nimotuzumab treatment enhanced cellular radiosensitivity in the clonogenic survival assay

**Figure 1:**
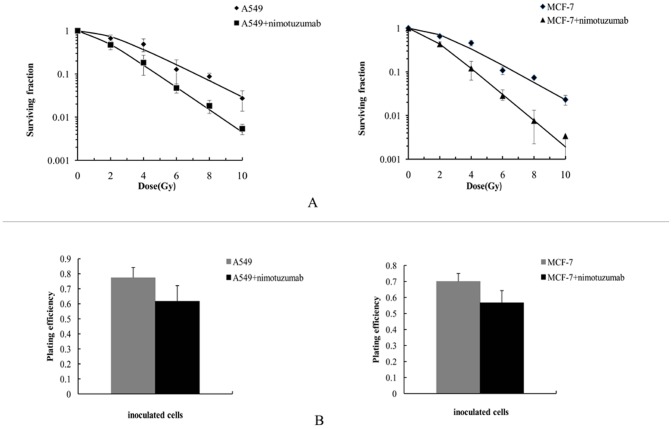
Dose-survival curves derived from clonogenic survival assay and the comparisons of plating efficiency. (A) A549 cells and MCF-7 cells were preincubated with and without 700 nM nimotuzumab for 24 h, and then radiated with doses of ionizing radiation between 2 and 10 Gy. After10–14 days, the colonies were stained and counted. Surviving fractions were calculated based on colony counts and plating efficiency. Data were shown as Mean ± SD from three independent experiments. Curves were fitted with SF = 1−(1−e^−D/D0^)^ N^. (B) Plating efficiency of A549 cells and MCF-7 cells. Plating efficiency = (mean number of colonies)/(the number of inoculated cells, which were pretreated with or without nimotuzumab). There were no statistical significances between nimotuzumab pretreated A549/MCF-7 cells and control, with P: 0.256, and 0.063, respectively.

To examine whether nimotuzumab enhanced the anticancer effect of ionizing radiation, we performed the clonogenic survival assay with A549 and MCF-7 cells (Figure 1; radiobiological parameters were summarized in Table 1). We did not find that the plating efficiency of both A549 and MCF-7 cells were significantly decreased after pretreatment with nimotuzumab (P: 0.256, and 0.063 respectively, shown as Figure 1B). Dose-survival curves indicated that pretreatment with nimotuzumab suppressed the clonogenic survival of both A549 cells and MCF-7 after varying doses of radiation (Figure 1A). The dose enhancement ratio was 1.36 and 1.47, respectively, which suggested that nimotuzumab was more effective in radiosensitizing MCF-7 cells than A549 cells in vitro.

**Table 1:** Radiobiological parameters of dose-survival curves (shown in Figure 1).

curve	D_0_	D_q_	N	SF_2_	SER
A549	2.27	2.00	2.41	0.73±0.13	
A549+nimotuzumab	1.67	0.99	1.81	0.48±0.11^a^	1.36
MCF-7	0.69	0.47	1.97	0.70±0.045	
MCF-7+nimotuzumab	0.47	0.42	2.44	0.43±0.057^b^	1.47

D_0_, mean lethal dose; D_q_, quasithreshold dose; N, extrapolation number; SF_2_, surviving fraction at 2Gy; SER: sensitivity enhancement ratio;

a : P: 0.003,

b : 0.002.

### Nimotuzumab inhibited EGFR activation after radiation

**Figure 2:**
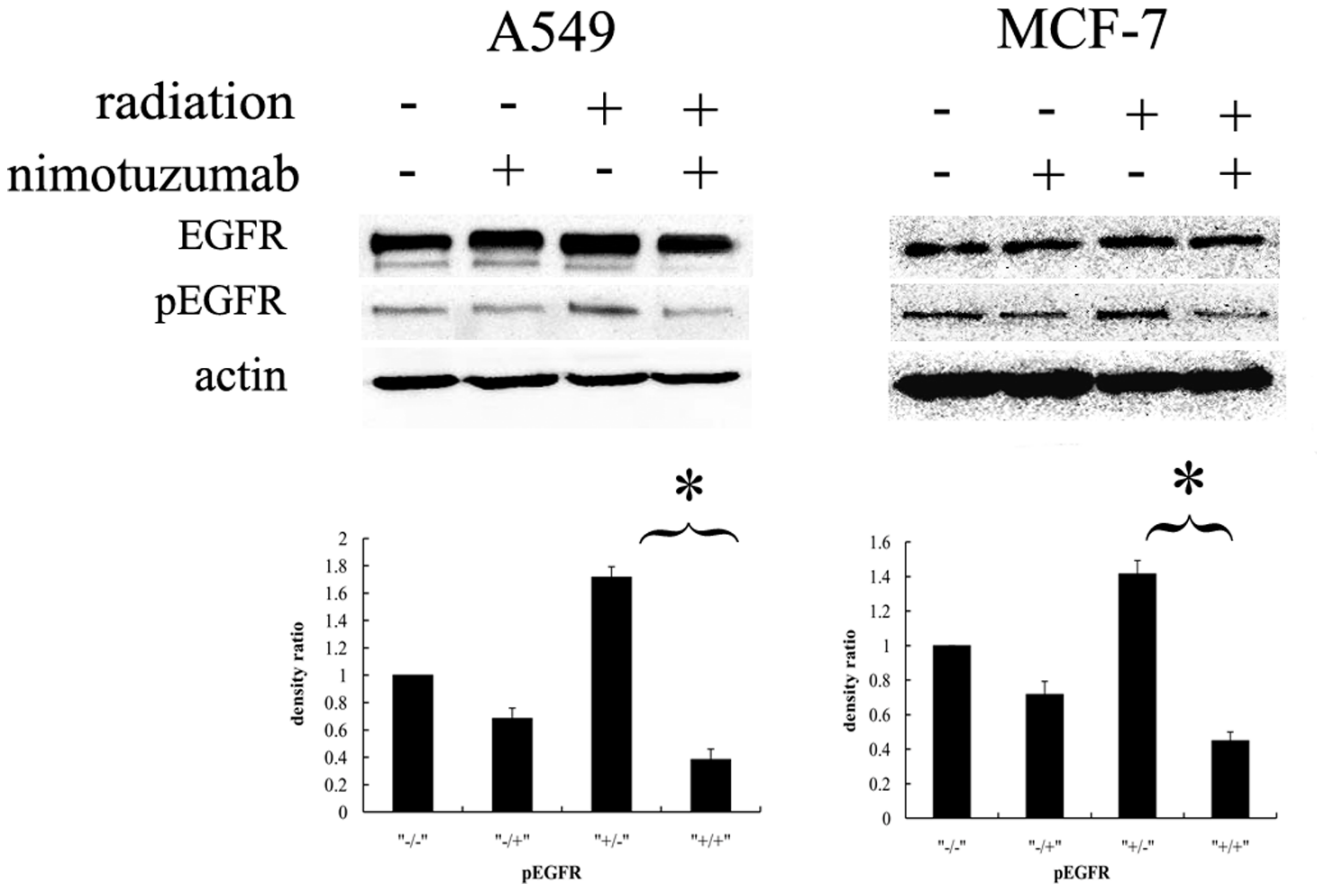
EGFR activation after treatment with nimotuzumab and radiation. A549 cells and MCF-7 cells were pretreated with or without nimotuzumab for 24 h before radiation with 4 Gy. Cells were harvested and lysed at 2 h after radiation. The lysates underwent electrophoresis and were incubated with antibodies against EGFR, pEGFR and β-actin. The column charts below are quantification of the bands based on densitometric analysis. Each bar represents the mean ± SD of relative density ratio to the control. * indicates significant difference (P<0.05).

To prove the capacity of nimotuzumab in inhibiting EGFR activation, the phosphorylation of EGFR at Tyr1173 was detected by western-blot (Figure 2). As it showed, radiation with 4Gy induced activated phosphorylation of EGFR in both A549 and MCF-7 cells. With the pretreatment of nimotuzumab, the levels of phosphorylated EGFR significantly decreased after radiation.

### Nimotuzumab increased cell apoptosis induced by radiation

**Figure 3:**
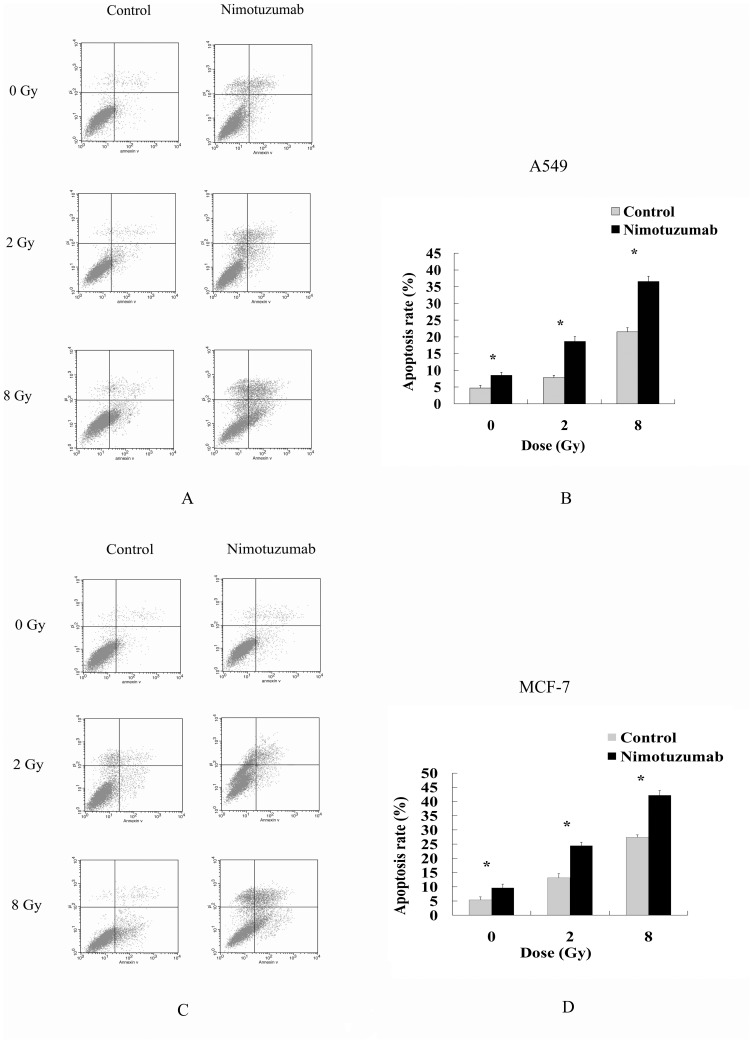
Apoptosis of A549 cells and MCF-7 cells induced by radiation and nimotuzumab. Cells were pretreated with and without 700 nM nimotuzumab for 24 h, and then irradiated with 0, 2 or 8 Gy. Fourty-eight hours after radiation, cells were harvested and cell apoptosis was examined by flow cytometry. One representative of three independent experiments of the two cells is shown (A, C). Comparisons of apoptosis rates of A549 cells and MCF-7 between the niomtuzumab pretreated and control groups are shown (B, D). Each bar represents the mean ± SD of apoptosis rate. * indicates significant difference (P<0.05).

Cell apoptosis rates were routinely analyzed to evaluate the anticancer effects of radiation. In this study, the apoptosis rates of A549 and MCF-7 treated with nimotuzumab were higher than those of control cells after receiving different doses of radiation (Figure 3). The result indicated that nimotuzumab increased the cytotoxic effect of ionizing radiation by inducing cell apoptosis.

### Pretreatment of nimotuzumab increased γ-H2AX formation in response to radiation

**Figure 4:**
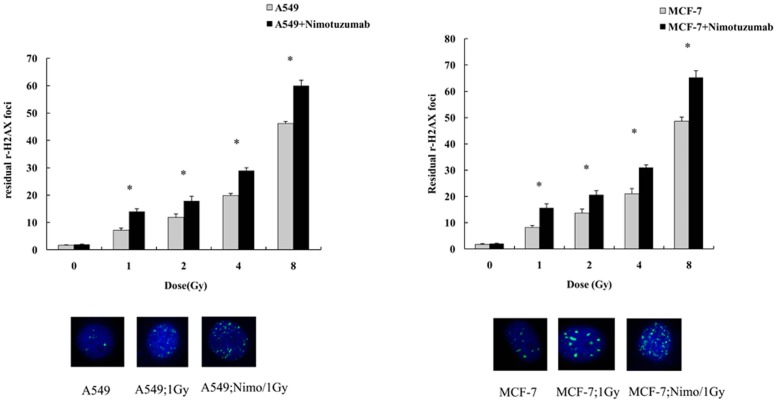
Quantification of residual γ-H2AX 24 h after radiation with different doses. A549 and MCF-7 cells were pretreated with or without 700 nM nimotuzumab for 24 h, and then were radiated with 1, 2, 4 and 8 Gy. Twenty four hours after radiation cells were fixed and incubated with γ-H2AX antibody conjugated with FITC. Each bar represents the mean ± SD of residual γ-H2AX foci per cell. For each data point at least 300 nuclei were evaluated. * indicates significant difference (P<0.05). The images below are representative nuclei with γ-H2AX foci under immunofluorescent microscopy, treated with 1Gy or 1Gy combined with nimotuzumab.

The quantification of γ-H2AX formation is a key indicator for radiation-induced DNA double strand breaks (DSBs). To evaluate the effects of combined treatment with nimotuzumab and radiation in DNA repair, we quantified the residual γ-H2AX foci 24 h after radiation with varying doses by confocal microscopy. We observed that pretreatment with nimotuzumab alone did not increase the γ-H2AX generation in both A549 and MCF-7 cells compared with control, while radiation treatment with different doses in both cells lines pretreated with nimotuzumab left more γ-H2AX formation after 24 h than cells with radiation alone (figure 4).

### Pretreatment of nimotuzumab regulated the DNA damage repair in response to radiation in A549 cells

Radiation-induced DNA damage repair is characterized by the expression of multiple DNA-damage repair related proteins. We therefore examined some of the related protein expressions and their activated forms under combined treatment with nimotuzumab and radiation in A549 cells.

**Figure 5:**
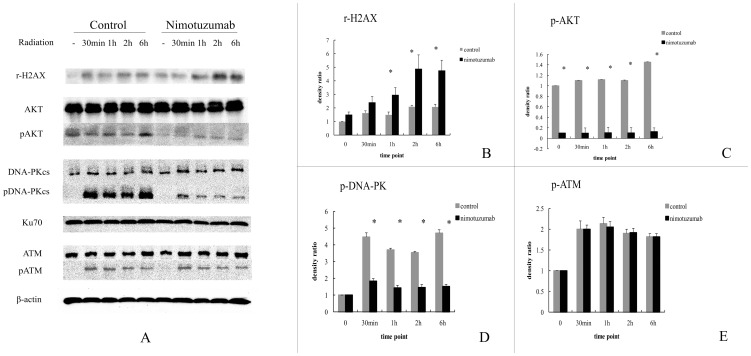
Time kinetics of DNA damage repair related proteins after radiation in A549 cells pretreated with nimotuzumab. (A) A549 cells pretreated with or without nimotuzumab for 24 h were irradiated with 4 Gy. Cells were harvested and lysed at indicated times. The lysates at indicated times underwent electrophoresis and were incubated with antibodies against γ-H2AX, AKT, pAKT, DNA-PKcs, pDNA-PKcs, Ku70, ATM, pATM and β-actin. (B–E) Quantification of bands based on densitometric analysis. The results are relative density ratio to the unirradiated control. Each bar represents the mean ± SD of three independent experiments. * indicates significant difference (P<0.05).

Firstly, we compared the kinetic change of these DNA damage repair related proteins in nimotuzumab treated and non-treated cells after exposure to radiation (see Figure 5). In this experiment, γ-H2AX in both cell groups showed a trend of time-dependent increase. In nimotuzumab pretreated group, the levels of γ-H2AX were significantly higher than those in the control group at 1 h, 2 h and 6 h (Figure 5B). DNA-PKcs, Ku70, ATM and AKT after radiation did not alter at each time point in both control and nimotuzumab pretreated groups. However, the phosphorylated form of DNA-PKcs at Thr2609 and the phosphorylated form of AKT at Thr308, which are activated forms of the two proteins induced by radiation, were expressed at lower levels in the nimotuzumab pretreated group than those in control at different time points. Unfortunately, both the levels of Phospho-DNA-PKcs and Phospho-AKT lacked the regular increasing tendency in correspondence with time (Figure 5C, D). The phosphorylated form of ATM at Ser1981 showed little alteration in both group, as well as at different time points (Figure 5E).

**Figure 6:**
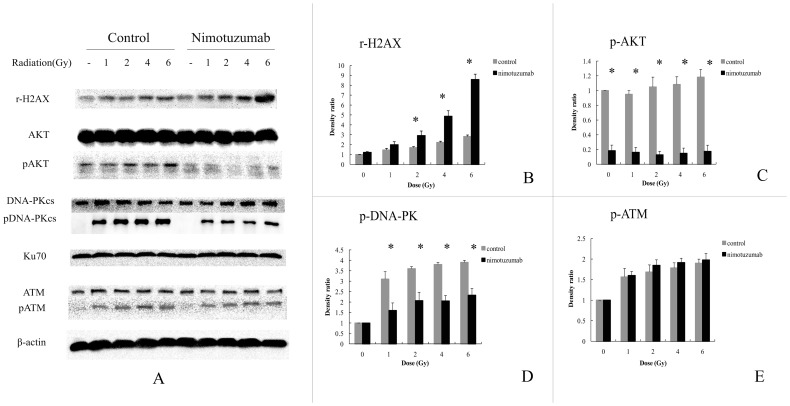
Effect of varying doses of radiation on DNA damage repair related proteins in A549 cells pretreated with nimotuzumab. (A) A549 cells pretreated with or without nimotuzumab were radiated with indicated doses. Two hours after radiation cells were harvested and lysed. The lysates underwent electrophoresis and were incubated with antibodies described in Figure 5. (B–E) Quantification of bands based on densitometric analysis. Each bar represents the mean ± SD of three independent experiments. * indicates significant difference (P<0.05).

Secondly, to investigate whether increasing the dose of radiation triggered more expression of DNA damage related proteins, and if nimotuzumab could inhibit the activation of DNA-PKcs and AKT, we radiated nimotuzumab pretreated A549 cells and control cells with varying doses. γ-H2AX showed a dose-dependent increase in both control and nimotuzumab pretreated group, and nimotuzumab pretreatment resulted in an significant increase of γ-H2AX (Figure 6B). Levels of DNA-PKcs, Ku70, ATM, pATM and AKT did not change despite the intense dose of radiation and pretreatment with nimotuzumab (Figure 6A, E). pDNA-PKcs and pAKT were obviously lower in nimotuzumab pretreated group than those in the control group, but did not show any significant variation at each dose in either group (Figure 6C, D).

## Discussion

Although monotherapy of anti-EGFR mAbs exhibits a limited efficacy in clinical trials, their combination with radiotherapy and/or chemotherapy has shown promising outcomes in treating advanced squamous cell cancer of head and neck, non small cell lung cancer and colorectal cancers [17]–[22]. The success of this combinational therapy is greatly attributed to the sensitizing effects of anti-EGFR mAbs to ionizing radiation and/or cytotoxic agents. Nimotuzumab, a humanized anti-EGFR mAbs, has demonstrated its unique capacity in generating radiosensitizing effects while causing mild toxicities of normal tissues in clinical trials [8], [12]. Although the anticancer effects of nimotuzumab have been confirmed in vitro and in vivo [23], the potential molecular mechanisms of nimotuzumab to radiosensitize cancer cells still need to be explored.

In the present study, we confirmed that the radiosensitivity of the cancer cell lines could be enhanced by pretreatment with nimotuzumab. Since nimotuzumab alone did not affect the colonies formation, it played a sensitizing but not additive role when combined with radiation. As the target of nimotuzumab, the activation of EGFR induced by radiation was obviously inhibited as it was expected. Furthermore, we demonstrated that nimotuzumab increased radiation-induced apoptosis of the two cell lines. Crombet-Ramos *et al* [23] reported that A431 cells incubated with imotuzumab for 48 h did not exhibit obvious apoptotic signs and concluded that the agent acted mainly as cytostatic instead of cytotoxic. In our study, however, we found that nimotuzumab increased the apoptosis rate of both A549 cells and MCF-7 cells fundamentally, which suggested that nimotuzumab as a therapeutic agent could induce cell apoptosis at least in vitro. As radiation combined with nimotuzumab resulted in a synergistic effect on cellular apoptosis greater than the sum of apoptosis induced by nimotuzumab and by radiation alone (i.e. a 1+1>2 effect), it is evidenced that nimotuzumab has the capacity of radiosensitizing these cancer cells. Furthermore, in our study, although nimotuzumab increased the apoptosis of cancer cells, it did not undermine the colonies formation. We speculated that the clonogenic cells of cancers were more resistant and difficult to be affected by nimotuzumab, and it was the proliferative cells that were induced to apoptosis by nimotuzumab.

Based on the above results, we then focused on the potential molecular mechanisms of nimotuzumab's radiosensitizing effects. It is well known that cancer radiosensitivity is mainly determined by the capacity of cancer cells to efficiently repair radiation-induced DNA damages. Therefore, we hypothesized that nimotuzumab might radiosensitize cancer cells by affecting the DSBs repair mechanism. γ-H2AX formation is a sensitive marker for DSB lesions and is routinely used to evaluate cellular radiosensitivity. More γ-H2AX foci means more DSBs left unrepaired. The quantified detection of γ-H2AX by immunofluorescence and immunoblot demonstrated that more DNA DSBs were left unrepaired after radiation in nimotuzumab pretreated cells, which ultimately led to the cell apoptosis, and showed a linear correlation with radiation doses or post-radiation time.

Next, we investigated how nimotuzumab inhibited the radiation-induced DSBs repair. DNA-PKcs is a critical protein involved in non-homologous end-joining repair of DNA DSBs. The activation of DNA-PKcs directly modulates the repair of radiation-induced DSBs. In the present study, the expression level of DNA-PKcs after radiation did not alter whether pretreated with nimotuzumab or not. But the phosphorylated form of DNA-PKcs at Thr2609, which is related to DSB repair and cellular radiosensitivity, strongly elevated after radiation, indicating a radioresistent attribute of A549 cells and significantly decreased in the nimotuzumab pretreatment group. Furthermore, we examined one of the regulatory subunits of DNA-PK complex Ku70. However, Ku70 did not alter in either group. Collectively, our results demonstrated that nimotuzumab inhibited the function of DNA-PKcs in DSB repair by suppressing its activation.

Toulany *et al* [24] reported that the EGFR-PI3K-AKT pathway was involved in the regulation of DNA-PKcs. They suggested that either EGFR tyrosine kinase inhibitor or AKT inhibitor abrogated radiation-induced DNA-PKcs phosphorylation at Thr2609 in K-RAS mutated tumor cells. Since nimotuzumab blocks EGFR downstream signaling, it is possible that nimotuzumab modulates the phosphorylation of DNA-PKcs through suppressing the activation of AKT. We found that the phosphorylated form of AKT at Thr308, which is the activated form associated with cell survival, highly increased in radiated A549 cells, but similar to phospho-DNA-PKcs (Thr2609), was totally inhibited after pretreatment with nimotuzumab. Thus, we concluded that nimotuzumab mediated its effects on DNA repair pathways via suppression of the PI3K-AKT pathway. Back to the cell apoptosis analysis, it confirmed that the inhibition of AKT activation by nimotuzumab is related to the promotion of cell apoptosis. The potential mechanism is via inhibiting the activation of DNA-PKcs, the repair of radiation induced DNA DSBs was prevented, which ultimately induced cell apoptosis.

ATM, an important protein participating in radiation-induced DNA damage repair, was studied to identify whether its expression or activity could be affected by pretreatment with nimotuzumab. ATM and its activated form, the phosphorylated form of ATM at Ser1981, did not change whether pretreated with nimotuzumab or not. Both DNA-PKcs and ATM phosphorylate γ-H2AX after ionizing radiation [24], [25]. In our study, since the activation of DNA-PKcs after radiation was inhibited by nimotuzumab through blocking PI3K-AKT pathway, the phosphorylation of H2AX may be due to the activity of ATM.

In summary, we showed that the anti-EGFR mAb nimotuzumab radiosensitizes cancer cells by inducing more apoptosis and unrepaired DSBs. The underlying mechanism of this radiosensitizing effect is related to the inhibition of DNA-PK involved DNA DSBs repair via the blockage of the PI3K-AKT pathway. Since nimotuzumab is an approved anti-EGFR mAb for therapeutic use in cancer treatment, exploring the precise anticancer mechanisms of nimotuzumab will help increase the efficacy of this agent in clinical practice.
